# Embedded Fiber Sensors to Monitor Temperature and Strain of Polymeric Parts Fabricated by Additive Manufacturing and Reinforced with NiTi Wires

**DOI:** 10.3390/s20041122

**Published:** 2020-02-19

**Authors:** Micael Nascimento, Patrick Inácio, Tiago Paixão, Edgar Camacho, Susana Novais, Telmo G. Santos, Francisco Manuel Braz Fernandes, João L. Pinto

**Affiliations:** 1 Department of Physics and i3N, University of Aveiro, Campus Universitário de Santiago, 3810-193 Aveiro, Portugal; tiagopaixao@ua.pt (T.P.); susana.novais@inesctec.pt (S.N.); jlp@ua.pt (J.L.P.); 2 UNIDEMI, Department of Mechanical and Industrial Engineering, Faculty of Science and Technology, Universidade NOVA de Lisboa, 2829-516 Caparica, Portugal; p.inacio@campus.fct.unl.pt (P.I.); telmo.santos@fct.unl.pt (T.G.S.); 3 CENIMAT/i3N, Department of Materials Science, Faculty of Science and Technology, Universidade NOVA de Lisboa, 2829-516 Caparica, Portugal; e.camacho@campus.fct.unl.pt (E.C.); fbf@fct.unl.pt (F.M.B.F.)

**Keywords:** optical fiber sensors, material extrusion, hybrid processes, temperature and strain monitoring

## Abstract

This paper focuses on three main issues regarding Material Extrusion (MEX) Additive Manufacturing (AM) of thermoplastic composites reinforced by pre-functionalized continuous Nickel–Titanium (NiTi) wires: (i) Evaluation of the effect of the MEX process on the properties of the pre-functionalized NiTi, (ii) evaluation of the mechanical and thermal behavior of the composite material during usage, (iii) the inspection of the parts by Non-Destructive Testing (NDT). For this purpose, an optical fiber sensing network, based on fiber Bragg grating and a cascaded optical fiber sensor, was successfully embedded during the 3D printing of a polylactic acid (PLA) matrix reinforced by NiTi wires. Thermal and mechanical perturbations were successfully registered as a consequence of thermal and mechanical stimuli. During a heating/cooling cycle, a maximum contraction of ≈100 µm was detected by the cascaded sensor in the PLA material at the end of the heating step (induced by Joule effect) of NiTi wires and a thermal perturbation associated with the structural transformation of austenite to R-phase was observed during the natural cooling step, near 33.0 °C. Regarding tensile cycling tests, higher increases in temperature arose when the applied force ranged between 0.7 and 1.1 kN, reaching a maximum temperature variation of 9.5 ± 0.1 °C. During the unload step, a slope change in the temperature behavior was detected, which is associated with the material transformation of the NiTi wire (martensite to austenite). The embedded optical sensing methodology presented here proved to be an effective and precise tool to identify structural transformations regarding the specific application as a Non-Destructive Testing for AM.

## 1. Introduction

Shape memory alloys (SMA) composites show their exceptional performance in adapting some physical parameters, such as shape, vibration, and impact resistance through a centralized control system [1,2]. However, a deformation of composite structures, resulting in the complex redistribution of stress states between matrix and reinforcement, is considered to be an important research topic that has been reported in the literature [2]. Thus, critical states reached by extreme deformation can be detected by using embedded sensors, which provide reliable information to the system. 

In the last years, important issues related with some constraints identified in SMA alloys, such as nickel–titanium (NiTi), have been reported: (1) The importance of indirect identification of the present phases and their transformations in a composite system incorporating SMA elements [3], (2) the incorporation of SMA allows to monitor the deformation/stress state of structural components but, if shape memory alloying elements are to be used to act as actuators, composite monitoring must be done by third sensory elements added. It is, therefore, important that these sensors can identify not only temperature variations and mechanical stress, but also, indirectly, the structural constituents present and the structural changes they undergo, especially given the nonlinearities of response that may exist in certain contexts [4].

The use of optical fiber sensors (OFS) for structural monitoring and detection of defects and temperature fluctuations in different materials has been suggested by several researchers [5,6,7]. There are many advantages associated with OFS technology, such as its reduced dimensions, immunity to electromagnetic interference, passivity, chemical inertness, multiplexing capability, nearly punctual sensing, and the capability to measure different parameters within one single optical fiber [8]. Regarding the specific application of OFS as a Non-Destructive Testing (NDT) for Additive Manufacturing (AM), different solutions can be applied as a complementary technique [9].

There are multiple OFS configurations, depending on the application, the required resolution, and/or sensitivity [8,10]. From all these possible configurations, fiber Bragg gratings (FBGs) are the most favorable solutions to NDT for AM based composite products. However, these sensors suffer from large cross-sensitivity, mainly strain, and temperature. To overcome this, a method based on using two different FBGs in two different fibers has shown to be the most reliable and easy technique to simultaneously monitor and discriminate these parameters [11]. Nevertheless, when it is intended to discriminate the parameters in embedded materials, such as polylactic acid (PLA) samples, this method can be a challenge, due to the need of having strain-free FBGs introduced inside other protective materials (for instance, a capillary tube), increasing the invasiveness on the sample [12].

To solve the need for internal discrimination of temperature and strain when monitoring their simultaneous variations, hybrid sensors comprising FBG and interferometric FP sensors can be fabricated, forming a cascaded optical sensor in which each element has different sensitivities. This way, the invasiveness inside the host material decreases, once only a single optical fiber is used to monitor the same point [13]. However, when this type of sensor is embedded in materials, an internal calibration for temperature and strain is needed, due to the mechanical stresses induced by the surrounding material [14,15]. Therefore, the use of OFS can be an important tool to assess and detect characteristic parameters in different types of materials, such as polymeric and/or SMA, depending on the sensing configuration used, behaving as an NDT. 

This work is about the use of AM (MEX) technology for the production of polymer matrix composite materials reinforced with previously functionalized NiTi wires. Three essential aspects of the application of this technology for the production of these composite materials were addressed: (1) The evaluation of the effect of the process (AM-MEX) on the properties of NiTi wires and their heat treatment (by monitoring the time and temperature to which it is subjected during production), (2) the evaluation of the mechanical and thermal behavior of the material in service (with the measurement of the stresses during tensile tests, evaluation of the adhesion of the matrix wires), (3) non-destructive inspection/material quality, using thermography and Joule effect on embedded NiTi wires to confirm disposition and whether heat-treatment (functionally graded materials) has been maintained. For that, an optical fiber sensing network based on FBGs and cascaded OFS was embedded in a 3D printed PLA matrix reinforced by NiTi wires to real-time monitor, temperature and strain shifts, in the PLA matrix, and temperature variations, which are associated to structural transformations in NiTi wires, during Joule heating of the NiTi wires and tensile cyclic load/unload.

## 2. Materials and Methods

### 2.1. Fiber Optic Sensors

Usually, an FBG sensor consists of a short segment of a single-mode optical fiber (SMF) with a photoinduced periodic modulation of the fiber core refractive index. When this device is illuminated by a broadband optical source, the reflected power spectrum shows a sharp peak, which is caused by interference of light with the planes of the grating and can be defined through Equation (1) [10]:

(1) $$\lambda_{B}=2n_{eff}\Lambda ,$$

where *λ_B_* is the so-called Bragg wavelength, *n_eff_* is the effective refractive index of the core mode, and Λ is the grating period. When the optical fiber is exposed to external parameters, such as temperature and strain, both *n_eff_* and Λ can be modified, resulting in a shift of the Bragg wavelength.

The sensor sensitivity towards a given parameter is obtained by monitoring the Bragg wavelength behavior while exposing the sensor to pre-determined and controlled conditions. In the case of a linear response, the sensitivity is provided by the slope of the obtained linear fit. The effects of temperature are accounted for in the Bragg wavelength shift by differentiating Equation (1):

(2) $$\Delta \lambda =\lambda_{B}\left(\frac{1}{n_{eff}}\frac{\partial n_{eff}}{\partial T}+\frac{1}{\Lambda }\frac{\partial \Lambda }{\partial T}\right)\Delta T=\lambda_{B}\left(\alpha +\xi \right)\Delta T=k_{T}\Delta T,$$

where *α* and *ξ* are the thermal expansion and thermo-optic coefficient of the optical fiber material, respectively [15]. By inscribing FBGs with different Bragg wavelengths, by changing the grating period, it is possible to get multiple temperature sensors within one single fiber. Thus, inspection and mapping of the sample temperature can be done by simultaneously monitoring the spectral variations of all sensors. This technique can be combined with other conventional methods, such as thermography analysis to produce a complete thermal analysis of a given sample material [16]. 

**Figure 1:**
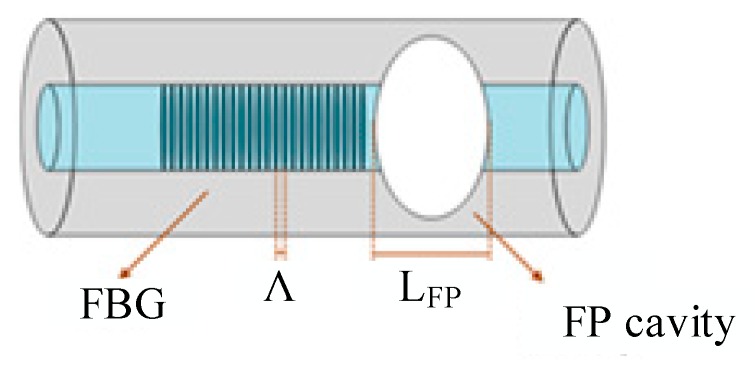
Diagram of the cascaded optical fiber sensor. L_FP_ represents the cavity length.

The sensing configuration that was employed to monitor the internal temperature and strain shifts on the PLA matrix consisted of cascaded optical fiber sensors, whose configuration scheme is shown in Figure 1. The simultaneous strain and temperature discrimination inside the PLA sample can be performed, combining the signals of the FBG sensor with the FP cavity interferometer, forming a cascaded optical fiber sensor. The FP cavity was fabricated by producing an air microbubble between a single-mode fiber (SMF 28e) and a multimode fiber (MMF, GIF625) [17]. To achieve point-of-care monitoring, the FBG was inscribed as close as possible to the FP interferometer. 

Assuming a linear response of the FBG to strain and temperature, the strain and temperature shifts (Δ*ε* and Δ*T*, respectively) are provided by:

(3) $$\Delta \lambda_{FBG}=k_{FBG_{\varepsilon }}\Delta \varepsilon +k_{FBG_{T}}\Delta T,$$

where *k_FBGε_*, and *k_FBGT_* are the strain and temperature sensitivities of the FBG, respectively, which were determined in the calibration procedure. 

The FP interferometer can also work as a strain and temperature sensor, where the wavelength shift, Δ*λ_FP_*, is given by:

(4) $$\Delta \lambda_{FP}=k_{FP_{\varepsilon }}\Delta \varepsilon +k_{FP_{T}}\Delta T,$$

where *k_FPε_* and *k_FPT_* are the strain and temperature sensitivities, respectively. Thus, the temperature and strain variations can be discriminated through the matrixial method, using Equations (3) and (4). If the sensitivity values are known, a sensitivity matrix for simultaneous measurement of strain and temperature can be obtained as:

(5) $$\left[\begin{array}{c} \begin{array}{c} \Delta \varepsilon \end{array}\\ \Delta T \end{array}\right]=\frac{1}{M}\left[\begin{array}{cc} k_{FP_{T}} & -k_{FBG_{T}}\\ -k_{FP_{\varepsilon }} & k_{FBG_{\varepsilon }} \end{array}\right]\left[\begin{array}{c} \Delta \lambda_{FBG}\\ \Delta \lambda_{FP} \end{array}\right],$$

where *M* = *k_FPT_* × *k_FBGε_* − *k_FPε_* × *k_FBGT_* is the determinant of the coefficient matrix, which must be non-zero for simultaneous measurement. Thus, internal discrimination of strain and temperature in the PLA matrix can be improved by combining the reflection spectra of this cascaded optical sensor. 

The main advantages of this process are different strain and temperature sensitivities between the two sensing elements, together with the use of a single fiber to monitor the same point, decreasing the invasiveness inside the PLA matrix composite. No extra-material integration is needed with this method. Moreover, the strain values obtained can be converted to displacement variations (Δ*L*), by multiplying the detected strain values by the sample length.

### 2.2. PLA Matrix and NiTi Wires

PLA is a thermoplastic material that has been widely used in components produced by AM, especially in Material Extrusion (MEX), due to the low melting point, good tensile stiffness and final surface quality. These properties potentiate the use of PLA as a matrix for the production of composite by MEX. 

NiTi ribbons (cross-section 3 × 1 mm^2^), were previously processed by localized heat-treatment at 400 °C during 10 min along a 20 mm segment, using Joule effect (21 A current). Previously, the NiTi ribbon was coated with black ink in order to establish an emissivity of around 0.95. All temperature measurements were performed with the infrared camera Fluke Ti400. After the local heat-treatment, these ribbons and the sensors were impregnated in the PLA matrix during the AM process as described in the next section.

### 2.3. Experimental Setup

**Figure 2:**
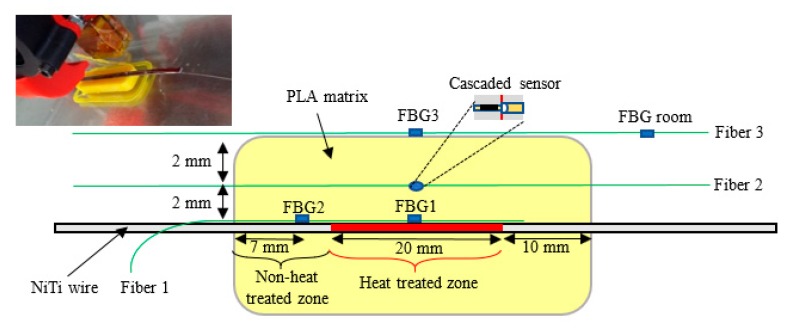
Experimental setup, sample cross-section view. Fiber 1 was embedded in the sample together with NiTi wire, while the 3D printing process was stopped for a few seconds. The same procedure was adopted to embed in the PLA matrix the cascaded sensor, recorded on fiber 2.

In order to assess the temperature and strain variations in samples of PLA matrix and verify the microstructural heterogeneity along NiTi wires, composed by heat-treated regions (marked in red) and the transition zones to the non-heat-treated zone, the experimental setup illustrated in Figure 2 with OFS was used. 

PLA samples were produced with the commercial BQ Prusa i3 3D printer, having a design comprising a cavity at the half-thickness in order to incorporate the NiTi wire and the optical fiber. After the fabrication, the model was sliced in the open-source software CURA. The feedstock was PLA and the print core had a nozzle with 1.2 mm of diameter. The layer height was set to have 0.5 mm, infill to 100%, and the print speed was 7 mm/s. At the half-thickness, the print was paused and the NiTi wire with the optical fiber 1 was incorporated in the PLA matrix. This process was repeated to embed the fiber 2 in the sample.

Two sets of samples of PLA + NiTi ribbon + sensors were prepared: One for the experiments on thermal cycling (no external mechanical load applied) and the other for the tensile tests (mechanical loading/unloading without any external thermal excitation).

The FBGs (length of ~3.0 mm each) were recorded in a photosensitive SMF (GF1, Thorlabs Inc., Newton, MA, USA), by the phase mask method. The inscribing system consists of focusing UV (266 nm) laser pulses originated by a pulsed Q-switched Nd:YAG laser system (LOTIS TII LS-2137U Laser, Minsk, Belarus) onto the SMF core by a plano-convex cylindrical lens (working length of 320 mm), passing through a phase mask. 

During the inscription of the FBGs, as well as throughout the experiments, the Bragg wavelengths were monitored by a single channel optical interrogator (sm125-500, Micron Optics Inc., Atlanta, GA, USA), operating at 1 Hz and wavelength accuracy of 1.0 pm. To read all the optical data at the same time, a 3 × 1 coupler was used.

Fiber 1 with the two FBGs were maintained strain-free, so they could detect temperature shifts of both heat-treated (FBG1) and non-heat-treated regions (FBG2). The cascaded sensor on fiber 2 simultaneously detected strain and temperature shifts on the PLA matrix. Externally, fiber 3, which has 2 FBGs (FBG3 and FBG room), was also placed in direct contact with the PLA sample surface to monitor external temperature shifts. The FBG room sensor was used out of the sample to monitor the room temperature variations, eliminating any possible external fluctuations.

The tensile tests were performed in a Shimadzu NG50KN, using a 50 kN load cell, a crosshead speed of 5 and 10 mm/min, and the maximum stroke of 6 % of the gauge length. 

### 2.4. Optical Fiber Sensing Calibrations

FBG1, FBG2, and cascaded optical sensors integration on the NiTi wire and in the PLA matrix, respectively, were done during the 3D manufacturing process. In this case, the fibers were fully embedded in the sample, thus presenting a more accurate response towards strain and temperature, when compared to external sensing devices. Before being embedded in the material, the fiber coatings were removed to minimize the intrusiveness of the sensing structures, presenting a total thickness of only 125 µm.

**Figure 3:**
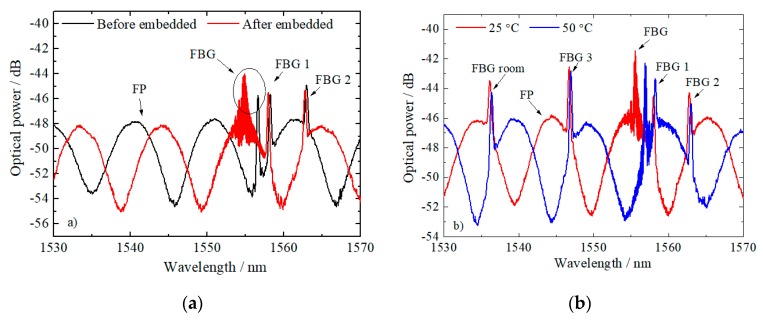
(**a**) Spectral response of the OFS after and before embedded in the polymeric sample. (**b**) Response of the cascaded optical sensor and FBGs after embedded on the sample at two different temperatures (25.0 °C and 50.0 °C).

Previous calibration of each sensing head towards each parameter was performed. Figure 3a,b shows the spectral response of the cascaded sensor and the FBGs after and before being embedded on the polymeric sample, and under two different temperatures, respectively. It is possible to observe the induced strain on the fiber sensors by the surrounding materials and a higher spectral change in the cascaded sensors, comparatively with the FBGs. Similar to other FBGs, the cascaded sensor (embedded in the PLA matrix) suffer higher induced strains.

Table 1 presents the strain and temperature sensitivities of the sensors before and after embedding in the PLA matrix and NiTi wire. From the internal strain and temperature calibrations, and according to the matrixial method (Equation (5)), a determinant value of 8.23 was obtained for the cascaded optical sensor embedded in the PLA matrix. 

The thermal calibrations after and before the sensors’ integration in the sample were performed in a thermal chamber (Model 340, Challenge Angelantoni Industrie, Massa Martana, Italy), between 15.0 °C and 60.0 °C, in steps of 5.0 °C. The strain characterization was performed using a micrometric translation stage between 0 με and 1000 με, in steps of 50 με.

## 3. Results and Discussion

### 3.1. Joule Heating of the NiTi Wire Tests

After the additive fabrication of the samples, they were cooled down to room temperature and then, a controlled intensity current was injected on the NiTi ribbon to heat it by Joule heating effect in the temperature range of 40 to 55 °C. 

**Figure 4:**
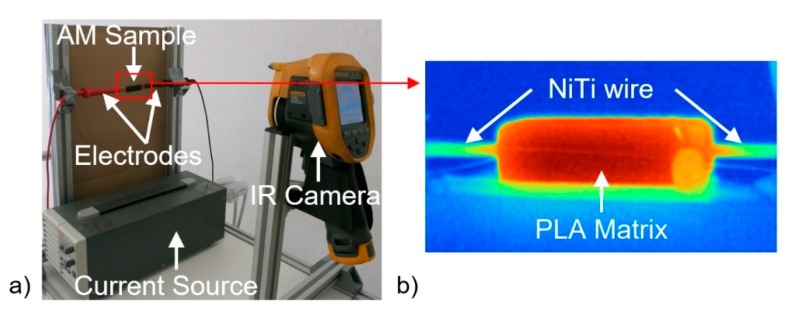
(**a**) Experimental setup used to perform the cycling Joule heating of the NiTi wire tests. (**b**) Inset of the external surface temperature measured by thermography in the sample.

The temperature variation on the heat-treated and non-heat-treated zones of the inserted NiTi wire was measured on the external surface by thermography (as can be seen in Figure 4), and internally by FBG1 and FBG2. The temperature and strain variations on the PLA matrix were monitored by the cascaded sensor.

In total, three cycling tests were performed in the sample. During the first cycle (test 1), currents of 2.12, 2.81, and 3.1 A were applied, followed by natural cooling to stabilize the sample temperature. For the tests 2 and 3, after applying the same three currents, a 4.0 A current was also used. 

**Figure 5:**
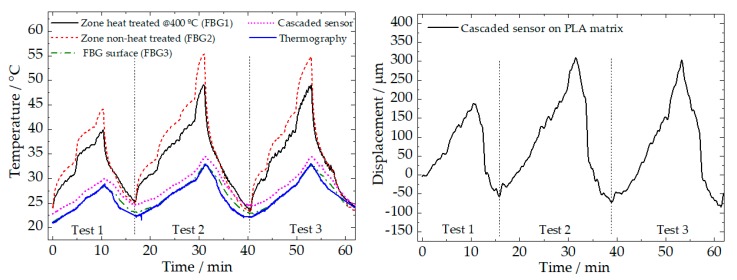
Temperature detected by all the sensing elements (**left**), and displacement sensed by the cascaded sensor in the PLA matrix (**right**), during the cyclic tests of heating by Joule effect, followed by natural cooling.

Figure 5 shows the results of the temperature variations detected by all the sensing elements used (FBGs, cascaded sensor, and thermography) at all the locations (surface, PLA matrix, and NiTi wire), where the cascaded sensor registers both strain and temperature variation in the PLA matrix.

The results show that a consistent deviation between the temperature of the external face of the PLA matrix (measured by thermography) and the temperature at the face of the NiTi wire can be observed. Moreover, a consistent deviation on the temperature measured at two different points of the NiTi wire is observed, with a significantly lower value in the heat-treated zone (more notorious when the higher current was applied), due the higher electrical conductivity of the heat-treated region. This effect could be assigned to the local reduction of structural defects (mostly dislocations) induced by the recrystallization associated to the localized heat-treatment. 

A small temperature difference (~1.5 °C) can be observed between the surface (as measured by thermography) and inside the PLA matrix, 2 mm below the surface (registered by the cascaded sensor). However, maximum displacement shifts of ~350 µm were detected, during tests 2 and 3. It is also observed that a successive contraction of the material after the heating/cooling cycles occurs. At the end of test 3, the contraction is ~100 µm, which may be due to the accommodation of the PLA material, indicating a good adhesion of the cascaded sensor to the surrounding material.

**Figure 6:**
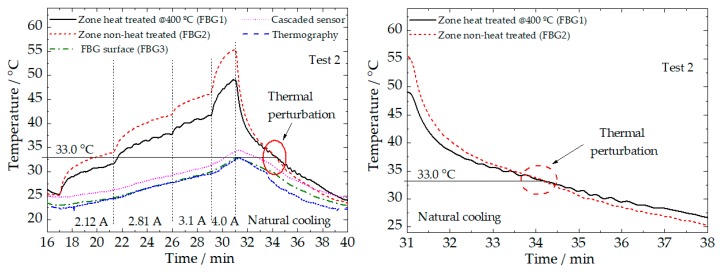
Temperature recorded by all the sensing elements during test 2 (**left**), and an inset during the natural cooling step (**right**), highlighting the thermal perturbation near the 33.0 °C.

The temperature recorded by all the sensing elements is highlighted in Figure 6. A thermal perturbation associated with a material phase transformation can be observed in the curves represented by the heat-treated and non-heat-treated zones (close to 33.0 ± 0.1 °C), during the natural cooling process. This perturbation may be assigned to a structural transformation (R-phase to austenite) taking place in the NiTi wire during cooling. 

The temperature shift for the two different regions of the NiTi wire (mostly remarkable during heating) may be assigned to different fractions of R-phase versus austenite (electrical resistivity of the R-phase is higher than that of the austenite). It is apparent that the optical sensors (FBG1 and FBG2) clearly identify the moment of phase transition in the two zones under study, for both cooling steps.

According to the sub-surface temperature variations detected by the surface FBG, there is a very good relationship with thermography values, although, and as expected, the temperature variations recorded internally by cascaded sensors in the PLA matrix are significantly higher (~2.0 °C difference). 

### 3.2. Tensile Tests

**Figure 7:**
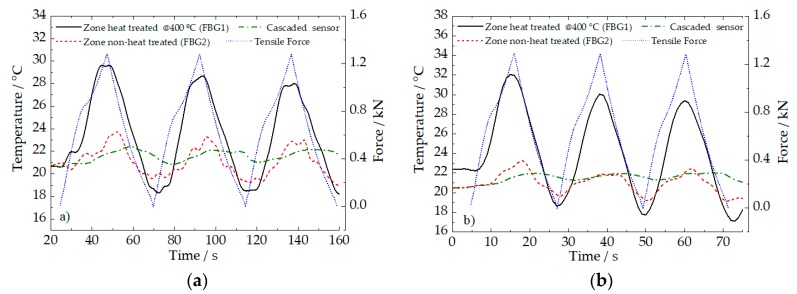

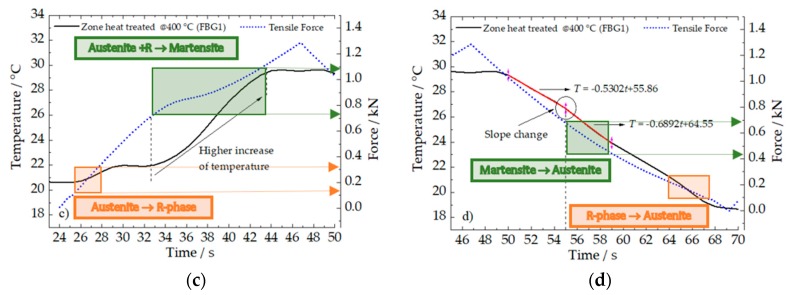
Temperature detected by the fiber sensors during the tensile cycling tests. (**a**) Tensile test at 5 mm/min. (**b**) Tensile test at 10 mm/min. (**c**) and (**d**) highlight the load and unload steps during the first cycle, at 5 mm/min, respectively.

Tensile cycling tests were performed on the NiTi wire with embedded sensors to study their thermal behavior regarding longitudinal deformation of the heat-treated and non-heat-treated regions. The sample was clamped on the extremities of the NiTi ribbon, by tensile test machine grips, typically used in tensile tests. Figure 7 shows the temperature results monitored by the fiber sensors placed in the heat-treated zone, non-heat-treated zone, and in the PLA matrix, while the tensile cycles were applied. 

In total, six cycles were applied to the sample: Three of them at 5 mm/min rate (Figure 7a) and the other three at 10 mm/min rate (Figure 7b). Basically, when the wire was submitted to the longitudinal deformation, a consequent exothermic and endothermic process could be observed during loading and unloading, respectively. At the end of each load/unload cycle, the temperature reached by the sample is lower than the temperature at the beginning of the corresponding cycle. 

Regarding the heat-treated and non-heat-treated zones on the NiTi wire, significative mean differences of 6.1 ± 0.1 °C, were detected. Notice that, on the non-heat-treated zone (FBG2) and the PLA matrix (hybrid sensor), the temperature peaks were reached by conduction, 4 and 10 s, respectively, after the heat-treated zone. 

As can be highlighted in Figure 7c, during loading, there are two load ranges where a steeper increase of temperature is observed: First, from 0.1 to 0.3 kN related to the stress-induced austenite R-phase transformation, and a second one, from 0.7 to 1.1 kN associated with the stress-induced austenite to martensite (B19’) transformation, both transformations are exothermal. The final step of the loading (above ~1.1 kN) corresponds to the elastic deformation of the stress-induced martensite, which, for this deformation rate, does not produce a significant amount of heat.

Analyzing the three cycles applied for each deformation rate, a successive decrease of maximum temperature is recorded by the optical sensors, being a consequence of the cooling associated to the reverse transformation (martensite to austenite) that takes place during the last step (downloading). The next cycle will then start from a lower temperature, so that the heating associated with the direct transformation (austenite to martensite) will not cause such an accentuated temperature increase as the one for the previous cycle. Additionally, due to this lower increase of temperature, the next downloading step will then go to a slightly lower temperature. This effect (decrease of the maximum temperature at the end of the loading step and of the minimum temperature at the end of the downloading) will be attenuated from one cycle to the next one.

Figure 7d, shows a zoom-in of the unload step during the first cycle at 5 mm/min. Near 55 s, a slope change may be observed in the temperature behavior. That is probably associated with the reverse stress-induced transformation (martensite to austenite) which is endothermal. At the final step of the unloading (below 0.3 kN, between 62 and 67 s), a new slope variation occurs, which is related to the R-phase→austenite transition (also endothermal).

For this particular application, the embedded sensing network proves to be a very effective tool to perform NDT, especially to identify and detect very localized temperature and strain variations, during operating processes in composite services. Comparing to other techniques (for instance thermography), this solution could detect a wide range of different parameters, such as structural transformations in SMA, and has very good reliability.

## 4. Conclusions

An OFS network was successfully developed and embedded during the 3D printing by AM of a PLA matrix reinforced by NiTi wire. Real-time monitoring of characteristic parameters, such as internal temperature and displacement shifts in the matrix and temperature variations in different treated zones of the wire allowed to use the OFS network as an NDT.

Joule heating experiments of NiTi wires were performed to assess changes to the sample temperature. The moments in which different currents were injected on the sample can be clearly proved and measured by all integrated fiber sensors. During the natural cooling, a thermal perturbation (structural transformation of R-phase to austenite) can be observed near 33.0 °C, and at the end of the cycling tests, a sample contraction of ~100 µm was detected on the PLA sample.

Regarding the tensile tests, the higher increase of temperature (exothermic behavior) arises when the applied force is between the 0.7 and 1.1 kN, on the heat-treated zone. During the unload step, a slope variation in the temperature behavior associated with the thermal-induced transformation in the heat-treated region (R-phase to austenite) was detected.

The embedded optical sensing methodology presented proved to be an effective, minimally invasive, and precise tool to identify materials’ structural transformations, revealing to be a suitable solution to be applied as an NDT for Additive Manufacturing.

## Figures and Tables

**Table 1:** Temperature and strain sensitivities of the cascaded optical sensor and FBGs obtained before and after embedding in the PLA matrix and NiTi wire, respectively.

***Cascaded Optical Sensor in PLA Matrix***				
Type of sensor	(*k_T_* ± 0.1) pm/°C		(*k_ε_* ± 0.1) pm/µε	
	*Before embedding*	*After embedding*	*Before embedding*	*After embedding*
FP (*L* = 115.4 ± 0.1 µm)	0.1	154.1	2.1	0.1
FBG	9.0	71.8	1.2	0.1
***FBGs in NiTi Wire***				
	(*k_T_* ± 0.1) pm/°C		(*k_ε_* ± 0.1) pm/µε	
	*Before embedding*	*After embedding*	*Before embedding*	*After embedding*
FBG1	9.5	9.5	1.1	-
FBG2	8.9	8.8	1.2	-
